# LncRNA RNCR3 promotes Chop expression by sponging miR-185-5p during MDSC differentiation

**DOI:** 10.18632/oncotarget.22906

**Published:** 2017-12-04

**Authors:** Wencong Shang, Zhenzhen Tang, Yunhuan Gao, Houbao Qi, Xiaomin Su, Yuan Zhang, Rongcun Yang

**Affiliations:** ^1^ State Key Laboratory of Medicinal Chemical Biology, Nankai University, Tianjin, China; ^2^ Key Laboratory of Bioactive Materials, Ministry of Education, Nankai University, Tianjin, China; ^3^ Department of Immunology, Nankai University School of Medicine, Nankai University, Tianjin, China

**Keywords:** myeloid-derived suppressor cells, RNCR3, miR-185-5p, Chop, epigenetic modification

## Abstract

Myeloid-derived suppressor cells (MDSCs) play a critical role in regulating immune responses in cancer and other pathological conditions. Mechanism(s) regulating MDSC differentiation and function is not completely clear, especially epigenetic regulation. In this study, we found that MDSCs express retinal non-coding RNA3 (RNCR3), and the expression in MDSCs is upregulated by inflammatory and tumor associated factors. RNCR3 may function as a competing endogenous RNA (ceRNA) to promote Chop expression by sponging miR-185-5p during MDSC differentiation. RNCR3 knockdown suppressed differentiation and function of MDSCs *in vitro* and *in vivo*. Quantitative RT-PCR showed that RNCR3 was negatively regulated by miR-185-5p in MDSCs. MiR-185-5p affected the expansion of MDSCs and reversed the effect of RNCR3 on MDSC differentiation and function through directly targeting Chop. Thus, our results suggest a RNCR3/miR-185-5p/Chop autologously strengthening network to promote MDSC differentiation and suppressive function in response to extracellular inflammatory and tumor-associated signals.

## INTRODUCTION

Myeloid-derived suppressor cells (MDSCs) have emerged as major regulators of immune responses in cancer and other pathological conditions [1–3]. In mice, MDSCs are identified by expressing both CD11b and Gr1 markers, and divided into two major subsets including polymorpho*nuclear* MDSC (PMN-MDSC) defined as CD11b^+^Ly6G^+^Ly6C^lo^ and monocytic MDSC (M-MDSC) defined as CD11b^+^Ly6G^-^Ly6C^hi^ [4, 5]. These cells express high levels of arginase-1(Arg-1), nitric oxide synthase 2 (NOS2/iNOS), NADPH oxidase 2 (NOX2) and prostaglandin endoperoxide synthase 2 (Ptgs2, also called COX2), resulting in production of nitric oxide (NO) and reactive oxygen species (ROS) [6, 7]. NO is labile and reacts with multiple compounds to produce many toxic and regulatory factors. ROS including hydrogen peroxide (H_2_O_2_), hydroxyl radical, and hypochlorous acid can damage proteins, lipids, and nucleic acids [8]. Whereas a high level of Arg-1 may cause elimination of key nutrition factors needed for T cell proliferation by depleting local environment L-arginine [9], L-cysteine [10] or tryptophan levels [11].

MDSCs are generated by sustained myelopoiesis during chronic inflammation, autoimmune diseases or cancer [1, 4, 12]. These MDSCs may also be induced *in vitro* by different cytokines including G-CSF, IL-6, GM-CSF, IL-1β, PEG2, VEGF and TNFα [13–17]. Notably, there exists remarkable difference in the constitution of subsets and suppressive function of MDSCs in response to different cytokines such as that GM-CSF plus IL-6 induced MDSCs have higher inhibitory activity than other MDSCs [18]. Multiple transcription factors and signaling pathways such as STAT3 (signal transducer of activator of transcription 3), C/EBPβ (CCAAT/enhancer binding protein β) and Chop (C/EBP homologous protein; encoded by Ddit3 and also known as Chop-10 and Gadd153) [19, 20] are involved in the regulation of MDSC differentiation and function [4, 13, 14]. However, differentiation and function of immune cells may be also epigenetically or post-translationally regulated. Emerging evidences show that long non-coding RNAs (lncRNAs) play critical roles in both developmental and differentiation processes by controlling gene expression. LncRNAs, that are longer than 200 nucleotides, are defined as a class of transcripts with no protein-coding potential. They can be either intergenic (between protein coding genes, long intergenic noncoding RNA (lincRNA)), intronic, natural antisense transcripts, or transcribed from divergent enhancers and promoters. So far, many lncRNAs have been shown to regulate the development and function of myeloid cells [21–23]. We have also demonstrated that lncRNA HOTAIRM1 can regulate peripheral blood cells to differentiate into dendritic cells (DCs) by sponging miR-3960 to regulate HOXA1 expression [24]. However, the effect(s) of lncRNA on the differentiation and function of MDSCs is very little understood. The understanding of specific molecular mechanism(s) responsible for MDSC differentiation and function would enable more precise therapeutics by targeting these cells.

RNCR3 (retinal noncoding RNA3, known as LINC00599 in human), a lncRNA transcribed from the intergenic regions of the genome and conserved in mammals [25], can regulate proliferation and/or function of different kinds of cells including neurons, oligodendrocyte, EC (endothelial cell), VSMC (vascular smooth muscle *cell)* and retinal endothelial cell [25–28]. Interestingly, RNCR3 knockdown also results in higher levels of inflammatory factors such as TNF-α, CCL2, and IL-6 in blood plasma, implying that RNCR3 may play a role in immune system [28]. In this study, we demonstrate that MDSCs express RNCR3, and this expression is significantly upregulated in inflammatory and tumor microenvironment. Importantly, we found that RNCR3 promotes MDSC differentiation and function by sponging miR-185-5p to release its target gene Chop. This may provide a novel regulatory mechanism for MDSC differentiation and suppressive function.

## RESULTS

### Tumor microenvironment upregulates expression of RNCR3 in MDSCs

**Figure 1:**
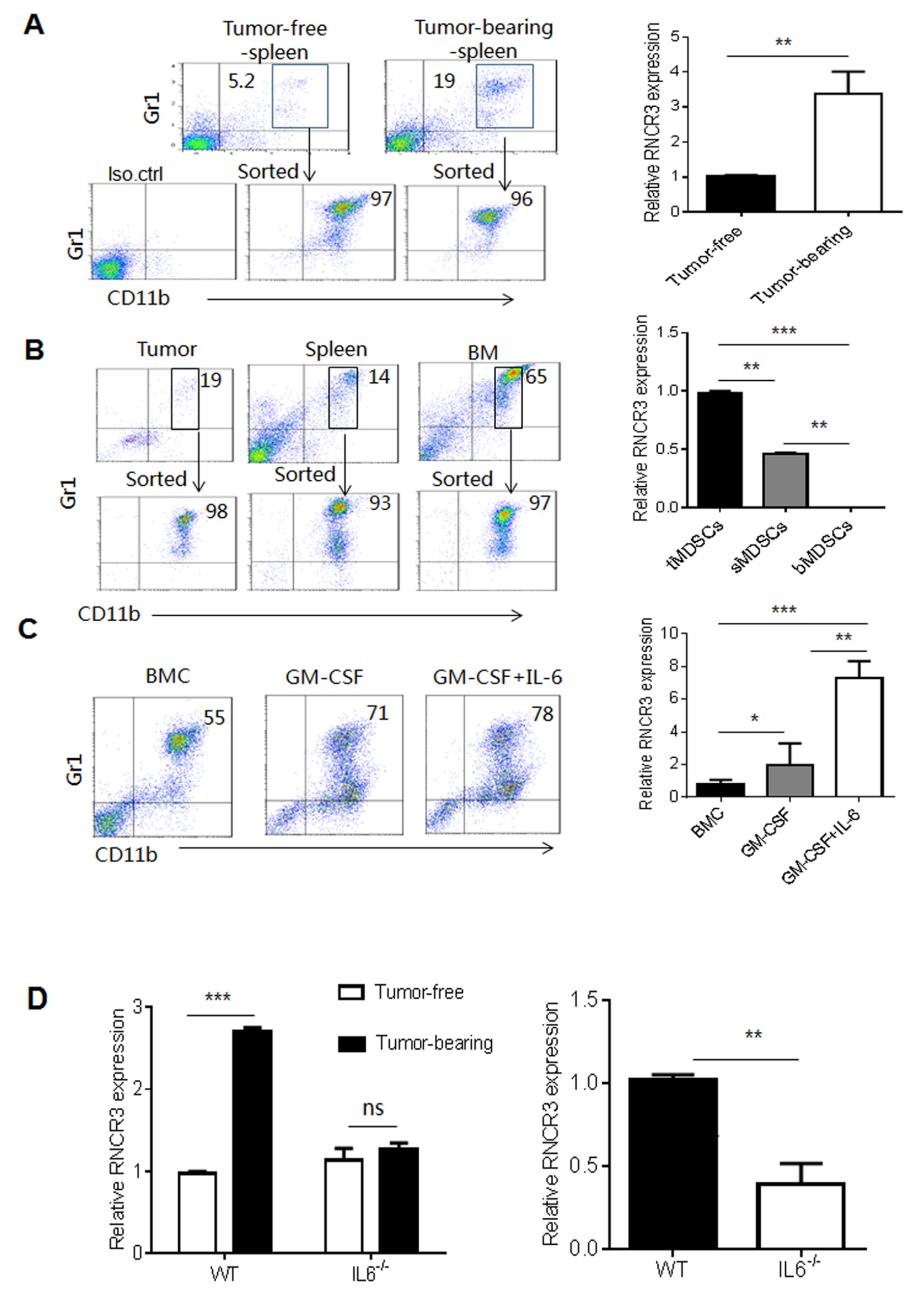
RNCR3 is upregulated in tumor microenvironment **(A)** Flow cytometric analyses (left) and RNCR3 qRT-PCR (right) of CD11b^+^Gr1^+^ cells in the spleen of tumor-free mice and B16 tumor-bearing mice. The MDSCs were sorted from the splenocytes of tumor-bearing mice and tumor-free mice. Iso.ctrl., antibody isotypic control. **(B)** Flow cytometric analyses (left) and RNCR3 qRT-PCR (right) of CD11b^+^Gr1^+^ cells in the tumor (tMDSC), spleen (sMDSC) and bone marrow (bMDSC) of B16 tumor-bearing mice. The MDSCs were sorted from tumor tissues, spleen and bone marrow (BM) of B16 tumor-bearing mice. **(C)** Flow cytometric analyses (left) and RNCR3 qRT-PCR (right) of CD11b^+^Gr1^+^MDSCs induced by GM-CSF or GM-CSF plus IL-6. Fresh CD11b^+^Gr1^+^ BMCs (BMC) were used as control. **(D)** QRT-PCR of RNCR3 in CD11b^+^Gr1^+^ MDSCs isolated from the BM of WT or IL-6^-/-^ tumor free and tumor-bearing mice (Left), and in CD11b^+^Gr1^+^MDSCs isolated from the BM of B16 tumor-bearing WT or IL-6^-/-^ mice (right). The data are representative of at least three separate experiments. ^*^P < 0.05, ^**^P < 0.01, ^***^p<0.005; Ns, no significance.

LncRNA RNCR3 (2998 bp) is mainly expressed in brain (www.lncrnadb.org). However, RNCR3 is also detected in EC (endothelial cell), VSMC (vascular smooth muscle *cell)* and retinal endothelial cell [25–28]. We here found that MDSCs also express RNCR3. RNCR3 could be detected using RT-PCR and confirmed by sequencing analyses in MDSCs (Supplementary Figure 1). This lncRNA is upregulated in aortic atherosclerotic lesions with hypoxia, oxidative stress, or inflammatory stress [28], which are shared by tumor environment. Indeed, RNCR3 expression in MDSCs could be upregulated in tumor microenvironment. MDSCs from the spleens of B16 tumor-bearing mice had higher levels of RNCR3 than those from the spleens of tumor-free mice (Figure 1A and 1B). The levels of RNCR3 were much higher in CD11b^+^Gr1^+^MDSCs isolated from tumor tissues than splenic CD11b^+^Gr1^+^MDSCs or bone marrow CD11b^+^Gr1^+^MDSCs (Figure 1B). To further explore factor(s) regulating RNCR3 expression in tumor microenvironment, we used different inflammatory factors to induce MDSCs from BMCs. We found that the levels of RNCR3 was much higher in CD11b^+^Gr1^+^ MDSCs after exposing to GM-CSF plus IL-6 as compared to those only exposed to GM-CSF (Figure 1C), indicating that IL-6 may be a critical factor in inducing RNCR3 expression. Since B16 melanoma cells do not produce IL-6 ([29] and not shown), IL-6 may be from tumor inflammatory environments not from B16 melanoma cells. Indeed, the levels of RNCR3 were markedly lower in the MDSCs isolated from bone marrow of IL-6 knockout (IL-6^-/-^) mice bearing B16 tumor than those in WT mice (Figure 1D). Taken together, these results indicate that tumor microenvironment factors such as IL-6 may promote the expression of RNCR3 during MDSC differentiation, implying that RNCR3 may play an important role in the differentiation and function of MDSCs.

### RNCR3 knockdown interrupts MDSC differentiation *in vitro*

**Figure 2:**
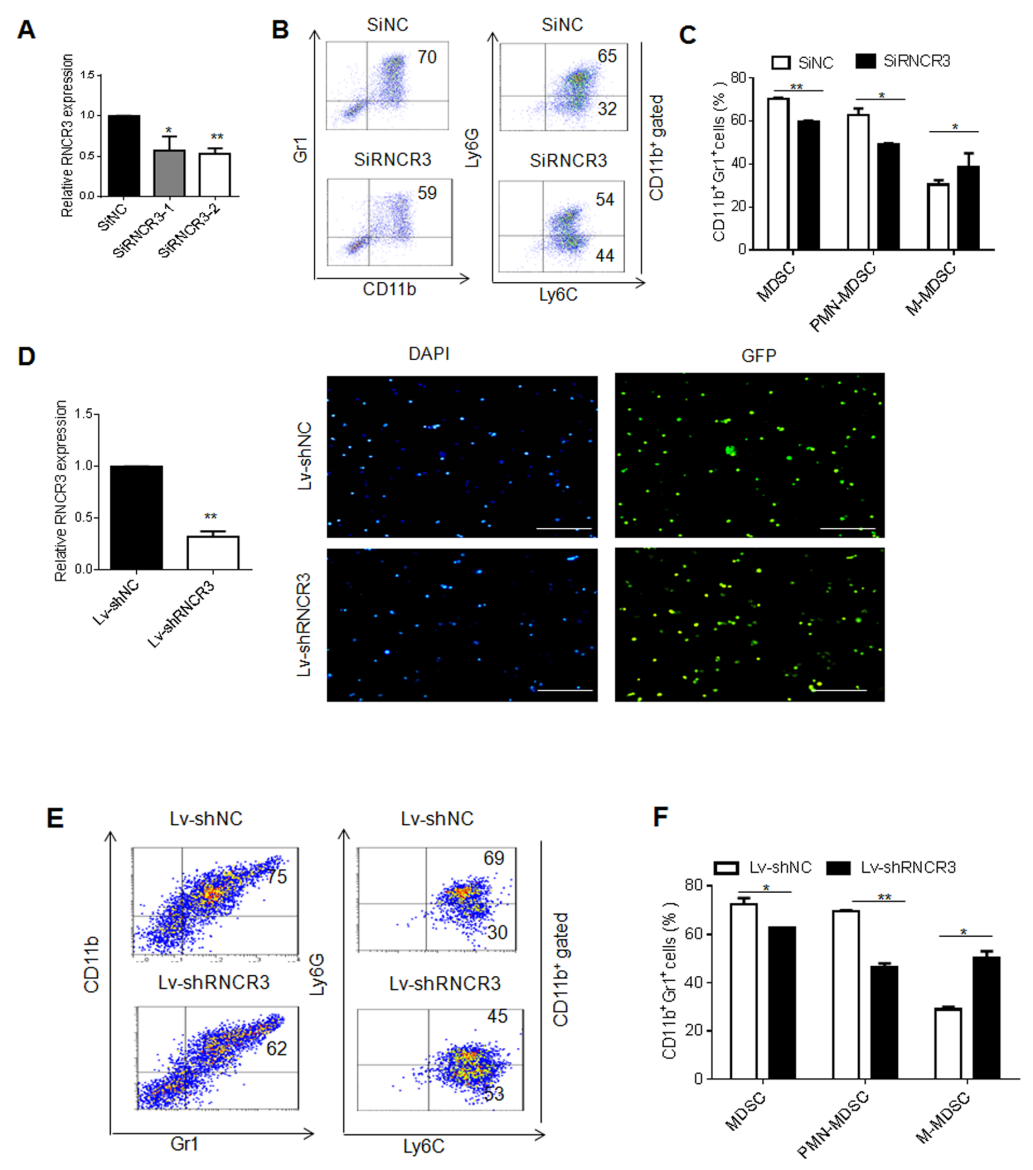
RNCR3 knockdown interrupts MDSC differentiation *in vitro* **(A)** QRT-PCR of lncRNA RNCR3 in MDSCs transfected with SiNC (siRNA control), SiRNCR3-1(RNCR3 siRNA 1) or SiRNCR3-2 (RNCR3 siRNA 2). **(B)** Flow cytometric analyses of MDSCs transfected with SiNC or SiRNCR3 (RNCR3 siRNA 2). **(C)** Statistical analyses of MDSCs transfected with SiNC or SiRNCR3 (RNCR3 siRNA 2). **(D)** QRT-PCR of RNCR3 in MDSCs transduced with control shRNA/lentiviruses (Lv-shNC) or RNCR3 shRNA/Lentiviruses (Lv-shRNCR3) (left), and fluorescence microscopy of lncRNA shRNA/lentiviruses transduced mouse MDSC (right). Blue (DAPI), nuclei; Green(GFP), lentivirus shRNA lentivirus. Rule bar =100μM. **(E)** Flow cytometric analyses of MDSCs transduced with control shRNA/lentiviruses (Lv-shNC) or RNCR3 shRNA/Lentiviruses (Lv-shRNCR3). **(F)** Statistical analyses of MDSCs transduced with control shRNA/lentiviruses (Lv-shNC) or RNCR3 shRNA/Lentiviruses (Lv-shRNCR3). The data are representative of at least three separate experiments. ^*^P < 0.05, ^**^P < 0.01, ^***^p<0.005; Ns, no significance.

To investigate the effect(s) of RNCR3 on the differentiation and function of MDSCs, we employed two kinds of knockdown methods including RNCR3 siRNA and RNCR3 shRNA. BMCs were transfected or transduced with RNCR3 siRNA or RNCR3 shRNA/lentivirus, and cultured *in vitro* for 4 days in the presence of GM-CSF and IL-6 to observe the effect of RNCR3 knockdown on the differentiation of MDSCs. We first designed two different RNCR3 siRNAs. Quantitative RT-PCR (qRT-PCR) revealed that the level of RNCR3 was reduced by RNCR3 siRNA transfection (Figure 2A). RNCR3 siRNA with stronger silencing efficiency was selected for subsequent assay. We found that RNCR3 knockdown decreased the percentage of Gr1^+^CD11b^+^ cells as compared to control (Figure 2B and 2C). Further analyses showed that the percentage of PMN-MDSCs was significantly reduced; whereas the proportion of M-MDSCs increased in these RNCR3 knockdown MDSCs (Figure 2B and 2C), indicating that RNCR3 is involved in the regulation of MDSC differentiation. We also prepared shRNA/lentiviruses with remarkably knockdown ability and over 90 % transduction rate (Figure 2D). Similar results could be also detected in the RNCR3 shRNA/lentivirus knockdown MDSCs (Figure 2E and 2F). These findings indicate that RNCR3 may regulate the differentiation of PMN-MDSCs and M-MDSCs subsets.

### RNCR3 knockdown weakens immunosuppressive function of MDSCs *in vitro*

**Figure 3:**
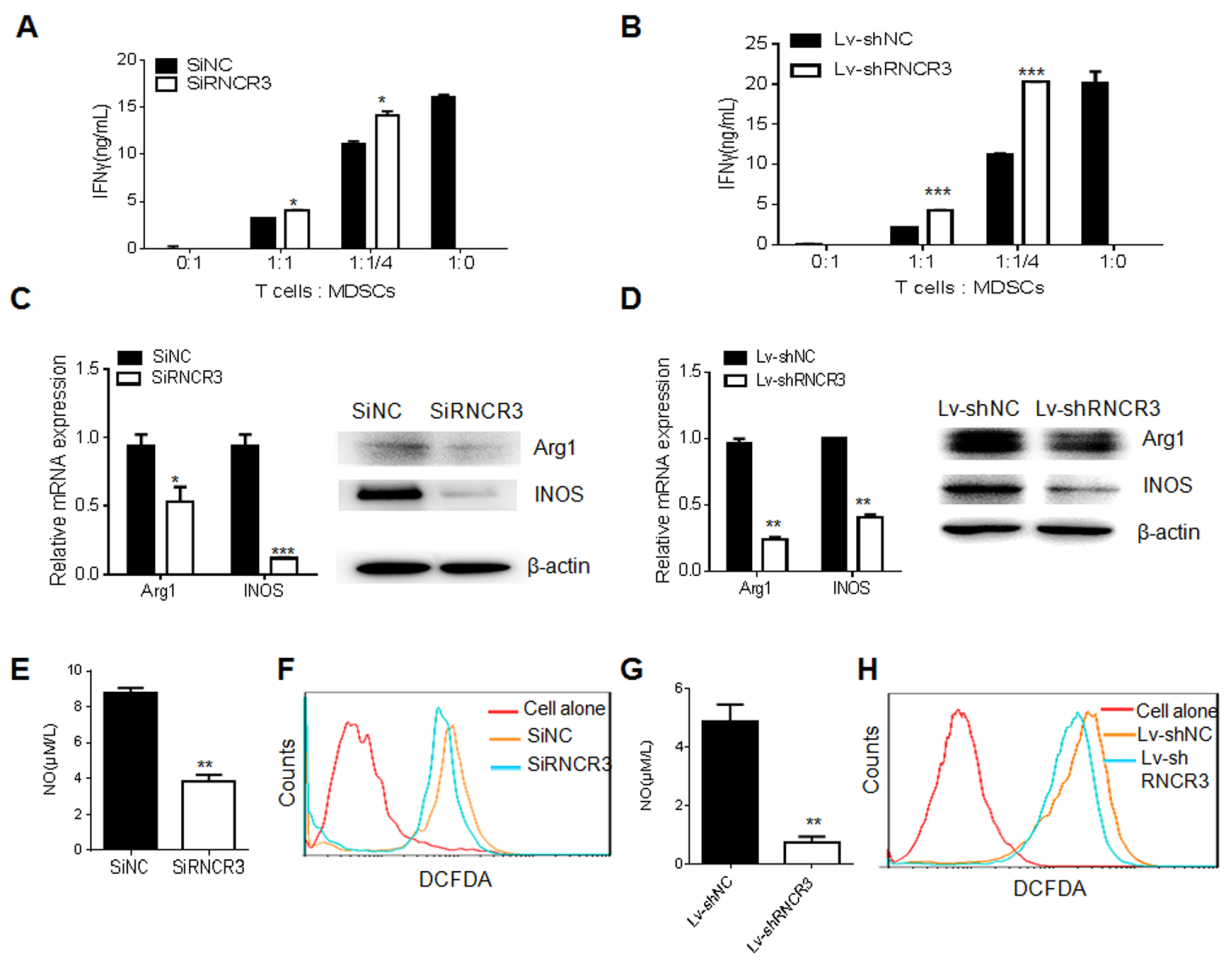
RNCR3 knockdown impairs immunosuppressive function of MDSCs *in vitro* **(A** and **B)** Suppressive capacity of RNCR3 siRNA (SiRNCR3) transfected (A) or RNCR3 shRNA/Lentivirus (Lv-shRNCR3) transduced (B) MDSCs. Activity of T cells was measured by their capacity to produce IFN-γ upon OVA-MHCI specific peptide stimulation. **(C** and **D)** QRT-PCR and immunoblotting of Arg-1 and iNOS in RNCR3 siRNA (SiRNCR3) transfected (C) or RNCR3 shRNA/Lentivirus (Lv-shRNCR3) transduced (D) MDSCs. **(E** and **G)** NO production in RNCR3 siRNA (SiRNCR3) transfected (E) or RNCR3 shRNA/Lentivirus (Lv-shRNCR3) transduced (G) MDSCs. **(F** and **H)** Flow cytometric analyses of ROS in RNCR3 siRNA (SiRNCR3) transfected (F) or RNCR3 shRNA/Lentivirus (Lv-shRNCR3) transduced (H) MDSCs. SiNC, siRNA control; Lv-shNC, control lentiviruses. The data are representative of at least three separate experiments. ^*^P < 0.05, ^**^P < 0.01, ^***^p<0.005; Ns, no significance.

We next determined the effect of RNCR3 on the immunosuppressive function of MDSCs. We co-cultured OVA-specific OT-I spleen cells with RNCR3 siRNA or RNCR3 shRNA knockdown MDSCs. RNCR3 knockdown MDSCs attenuated the suppression of MDSCs on IFN-γ secretion of OT-I T cells (Figure 3A and 3B). The suppression of MDSCs on T cells mainly depends on Arg-1, iNOS, NOX2 and their products [6, 7]. QRT-PCR and Western blotting showed that RNCR3 knockdown significantly decreased the expression of Arg-1 and iNOS in MDSCs (Figure 3C and 3D). RNCR3 silencing MDSCs had also lower levels of NO (Figure 3E and 3G) and ROS (Figure 3F and 3H) than control MDSCs. These results suggest that RNCR3 may regulate the immunosuppressive function of MDSCs.

### RNCR3 knockdown suppresses differentiation and function of MDSCs *in vivo*

**Figure 4:**
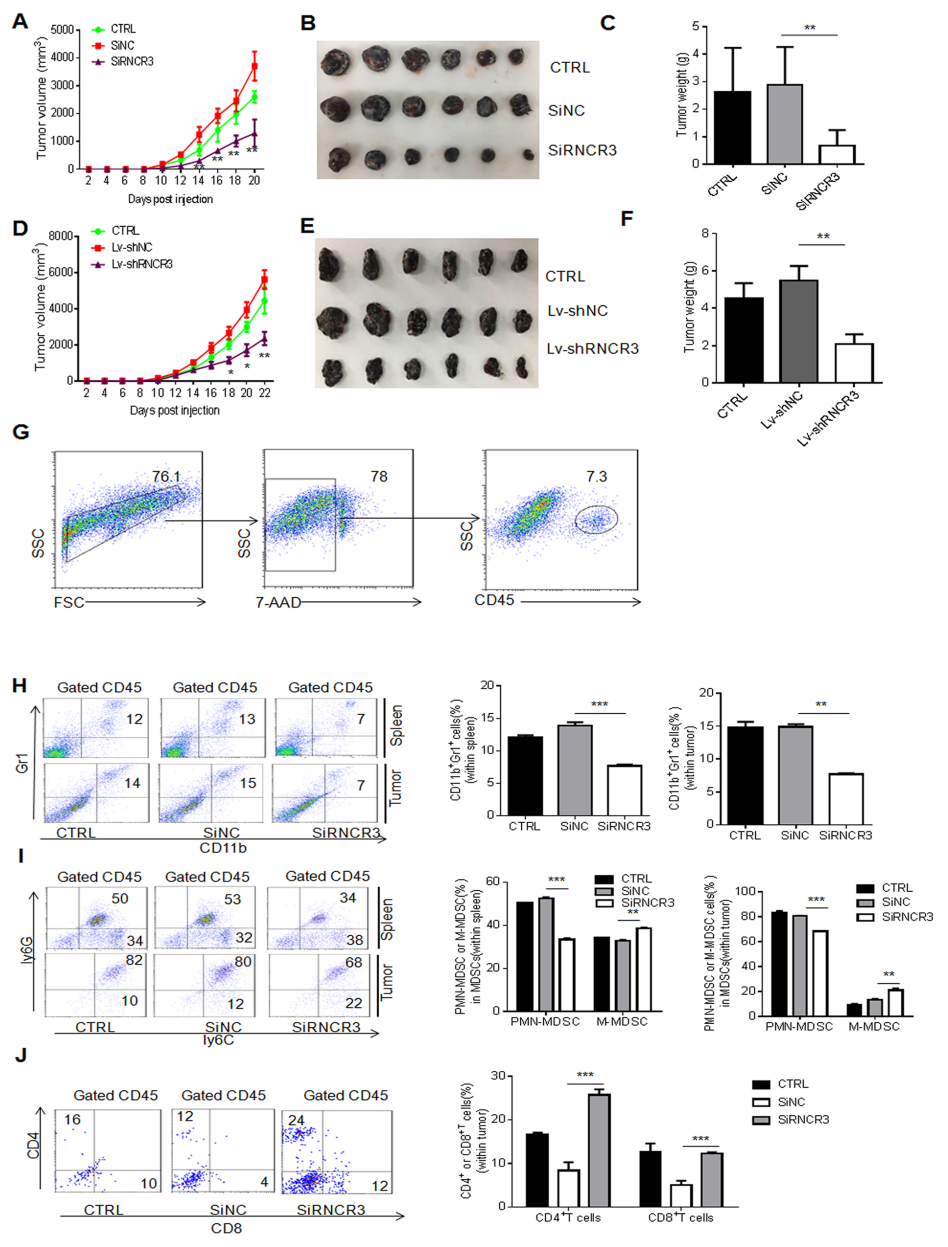
RNCR3 knockdown affects differentiation and immunosuppressive function of MDSCs *in vivo* **(A-F)** Tumor growth (A, D), tumor size (B, E) and tumor weight (C, F) in C57/BL6 mice bearing B16 tumors (N=6), which were injected with CD45.1^+^MDSCs transfected with SiRNCR3 (RNCR3 siRNA) (A, B, C) or transduced by shRNA (Lv-shRNCR3) (D, E, F). SiNC, siRNA control; Lv-shNC, control lentivirus; CTRL, without MDSC injection. **(G)** Gated strategies for tumor immune cells. **(H** and **I)** Flow cytometric and statistical analyses of MDSCs (H) and their subpopulations (I) in the spleen and tumor from mice injected with CD45.1^+^ MDSCs transfected with SiNC or SiRNCR3. **(J)** Flow cytometric and statistical analyses of CD4^+^ or CD8^+^ cells in tumor from mice injected with or without CD45.1^+^MDSCs transfected with SiNC or SiRNCR3. The data are from three separate experiments. ^*^P < 0.05, ^**^P < 0.01, ^***^p<0.005; Ns, no significance.

To further determine the effect of RNCR3 on the differentiation and function of MDSCs, we employed a murine B16 melanoma model. RNCR3 knockdown MDSCs were injected into mice after inoculating B16 tumor cells. The tumor growth was monitored at the indicated time. Mice injected with MDSCs transfected with RNCR3 siRNAs or transduced with RNCR3 shRNA/lentivirus showed decreased tumor growth, tumor size and tumor weight (Figure 4A-4F) as compared to control. In addition, we also found mice injected with MDSCs transfected with RNCR3 siRNA had a decreased CD11b^+^Gr1^+^ cells and CD11b^+^ly6G^+^ ly6C^low^ subsets in the spleen and tumor (Figure 4G-4I), supporting the involvement of RNCR3 in the differentiation of MDSCs. The percentages of CD4^+^ and CD8^+^ cells were increased in the tumor of the mice injected with MDSCs transfected with RNCR3 siRNA as compared to control (Figure 4J), indicating the reduced suppressive ability in RNCR3 knockdown MDSCs. These changes of immune cell populations in the spleen and tumor were also found in mice injected with MDSCs transduced with RNCR3shRNA/lentiviruses (Supplementary Figure 2). Although MDSCs have direct cytotoxicity on the tumor cells [30], but this effect is very weak and no difference between RNCR3 knockdown and control treated MDSCs (not shown), eliminating the direct antitumor effect of RNCR3-treated MDSCs. Taken together, these results indicate that RNCR3 knockdown interrupts the differentiation of MDSCs and weakens the immunosuppressive activity of MDSCs *in vivo*.

### Regulation of RNCR3 is through a reciprocal interaction with miR-185-5p

**Figure 5:**
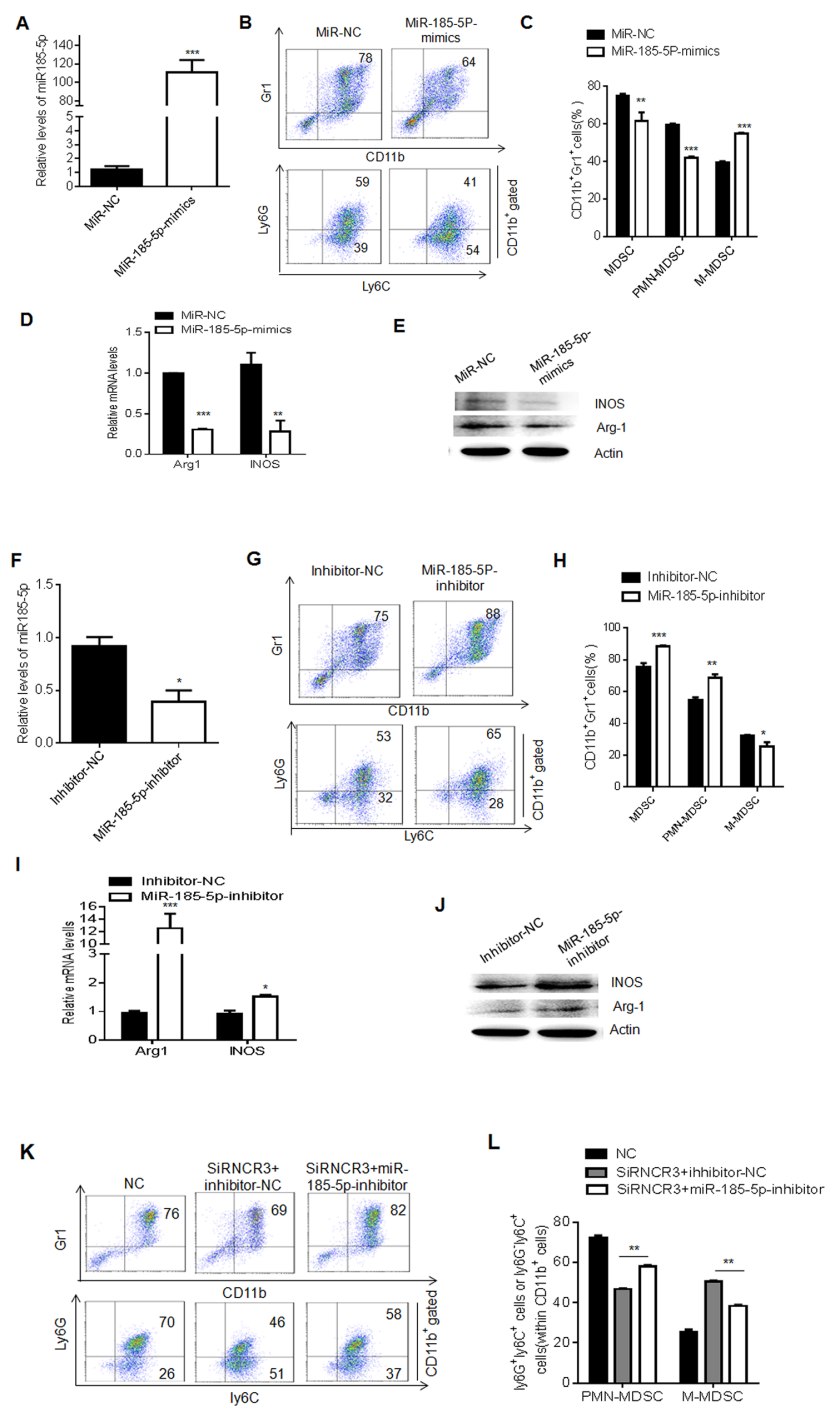
MiR-185-5p regulates differentiation and function of MDSCs **(A)** QRT-PCR of miR-185-5p in MDSCs transfected with miR-185-5p mimics or MiR-NC (mimics control) in the presence of GM-CSF and IL-6. **(B** and **C)** Flow cytometric (B) and statistical analyses (C) of the MDSCs transfected with miR-185-5p mimics or NC. **(D** and **E)** QRT-PCR (D) and Western blot (E) of iNOS and Arg-1 in MDSCs transfected with miR-185-5p mimics control (NC) or miR-185-5p mimics. **(F)** QRT-PCR of miR-185-5p in the MDSCs transfected with miR-185-5p inhibitor or inhibitor control (NC). **(G** and **H)** Flow cytometric (G) and statistical analyses (H) of MDSCs transfected with miR-185-5p inhibitor or inhibitor control (NC). **(I** and **J)** QRT-PCR (I) and Western blot (J) of iNOS and Arg-1 in MDSCs transfected with miR-185-5p inhibitor control (NC) or miR-185-5p inhibitor. **(K** and **L)** Flow cytometric (K) and statistical analyses (L) of MDSCs transfected with RNCR3 siRNA and miR-185-5p inhibitor (siRNCR3+miR-185-5p inhibitor) or inhibitor control (siRNCR3+inhibitor-NC). NC, siRNA and inhibitor control. The data are representative of at least three separate experiments. ^*^P < 0.05, ^**^P < 0.01, ^***^p<0.005; Ns, no significance.

LncRNAs could act as ceRNAs by interacting with miRNAs to regulate cell function and differentiation. A previous study has shown that RNCR3 can function as a ceRNA by binding to miR-185-5p, and miR-185-5p mimics significantly decrease the level of RNCR3 [28]. Similar phenomenon was also observed in MDSCs. We first found that miR-185-5p could be downregulated by GM-CSF plus IL-6 (Supplementary Figure 3A), which was inverse to RNCR3 (Figure 1C), suggesting a potential correlation between RNCR3 and miR-185-5p. While miR-185-5p mimics was transfected into BMCs, the level of RNCR3 was significantly downregulated (Supplementary Figure 3B and 3C); Conversely, knockdown RNCR3 increased miR-185-5p level (Supplementary Figure 3D). Since RNCR3 may interact with miR-185-5p, we next determined whether miR-185-5p was involved in the MDSC differentiation. BMCs were transfected with miR-185-5p mimics or miR-185-5p inhibitor in the presence of GM-CSF and IL-6. The level of miR-185-5p was significantly higher in MDSCs transfected with miR-185-5p mimics (Figure 5A). After induction for 4 days, miR-185-5p mimics reduced the percentage of CD11b^+^Gr1^+^MDSCs and the proportion of PMN-MDSCs, but the proportion of M-MDSCs in CD11b^+^cells increased as compared to those transfected with mimics control (Figure 5B and 5C). Furthermore, mRNA and protein levels of Arg-1 and iNOS also decreased in those MDSCs transfected with miR-185-5p mimics (Figure 5D and 5E). In contrast, miR-185-5p inhibitor significantly downregulated the level of miR-185-5p (Figure 5F), increased the percentage of CD11b^+^Gr1^+^MDSCs and PMN-MDSCs, and decreased M-MDSCs as compared to those transfected with inhibitor control (Figure 5G and 5H). MiR-185-5p inhibitor transfected MDSCs also increased Arg-1 and iNOS (Figure 5I and 5J). Thus, our data demonstrate that miR-185-5p may regulate the differentiation and function of MDSCs. We next further determined whether RNCR3 regulated the differentiation of MDSCs by binding to miR-185-5p. We found that miR-185-5p inhibitor could partially recover reduced MDSCs and PMN-MDSCs, which was caused by RNCR3 knockdown (Figure 5K and 5L), further supporting that miR-185-5p may interact with RNCR3 to affect the differentiation and function of MDSCs. Taken together, these results demonstrate that RNCR3-mediated differentiation and function of MDSCs is through miR-185-5p.

### miR-185-5p directly targets Chop

**Figure 6:**
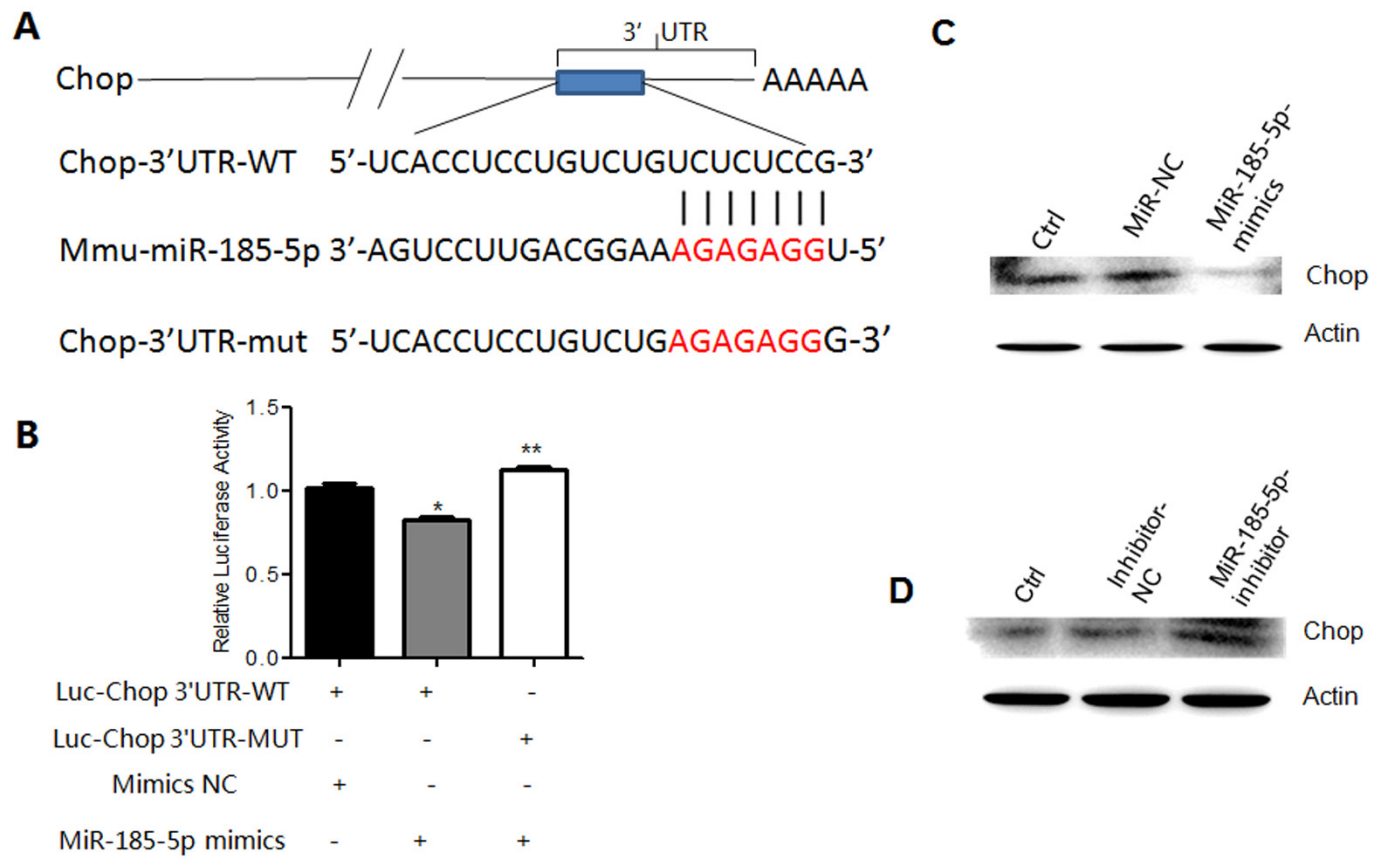
MiR-185-5p directly targets Chop **(A)** Predicted paring of miR-185-5p to the 3’-UTR of Chop and the mutant site (Red) of Chop 3’-UTR in pSiCHECK^™^-2 luciferase reporter vector. **(B)** Dual-luciferase reporter assay of 293T cells co-transfected with Chop 3’-UTR-WT or Chop 3’-UTR-Mut and miR-185-5p mimics or mimics control (NC). **(C)** Western blot of Chop in MDSCs transfected with mimics control (miR-NC) or miR-185-5p mimics. Untransfected MDSCs as control. **(D)** Western blot of Chop in MDSCs transfected with inhibitor control (inhibitor-NC) or miR-185-5P inhibitor. Untransfected MDSCs as control. ^*^P < 0.05, ^**^P < 0.01, ^***^p<0.005; Ns, no significance.

We next investigated how miR-185-5p to regulate the differentiation and function of MDSCs. Generally, miRNAs regulate cell processes by controlling the expression of their target genes. We first predicted the target gene(s) of miR-185-5p by Targetscan and miRanda, and found that Chop was a potential target gene (Figure 6A). To demonstrate that Chop is a target of miR-185-5p, we constructed luc-Chop 3’UTR and luc-Chop 3’UTR mutant (mutated on the putative miR-185-5p sites) vectors (Figure 6A). Luciferase assays showed that miR-185-5p mimics inhibited luciferase activity of luc-Chop 3’UTR but not luc-Chop 3’UTR mutants (Figure 6B), suggesting that miR-185-5p may bind with Chop 3’UTR. Further studies showed that miR-185-5p mimics also inhibited the expression of Chop (Figure 6C); whereas miR-185-5p inhibitor promoted the expression of Chop (Figure 6D). Thus, we demonstrate that Chop is a target of miR-185-5p.

**Figure 7:**
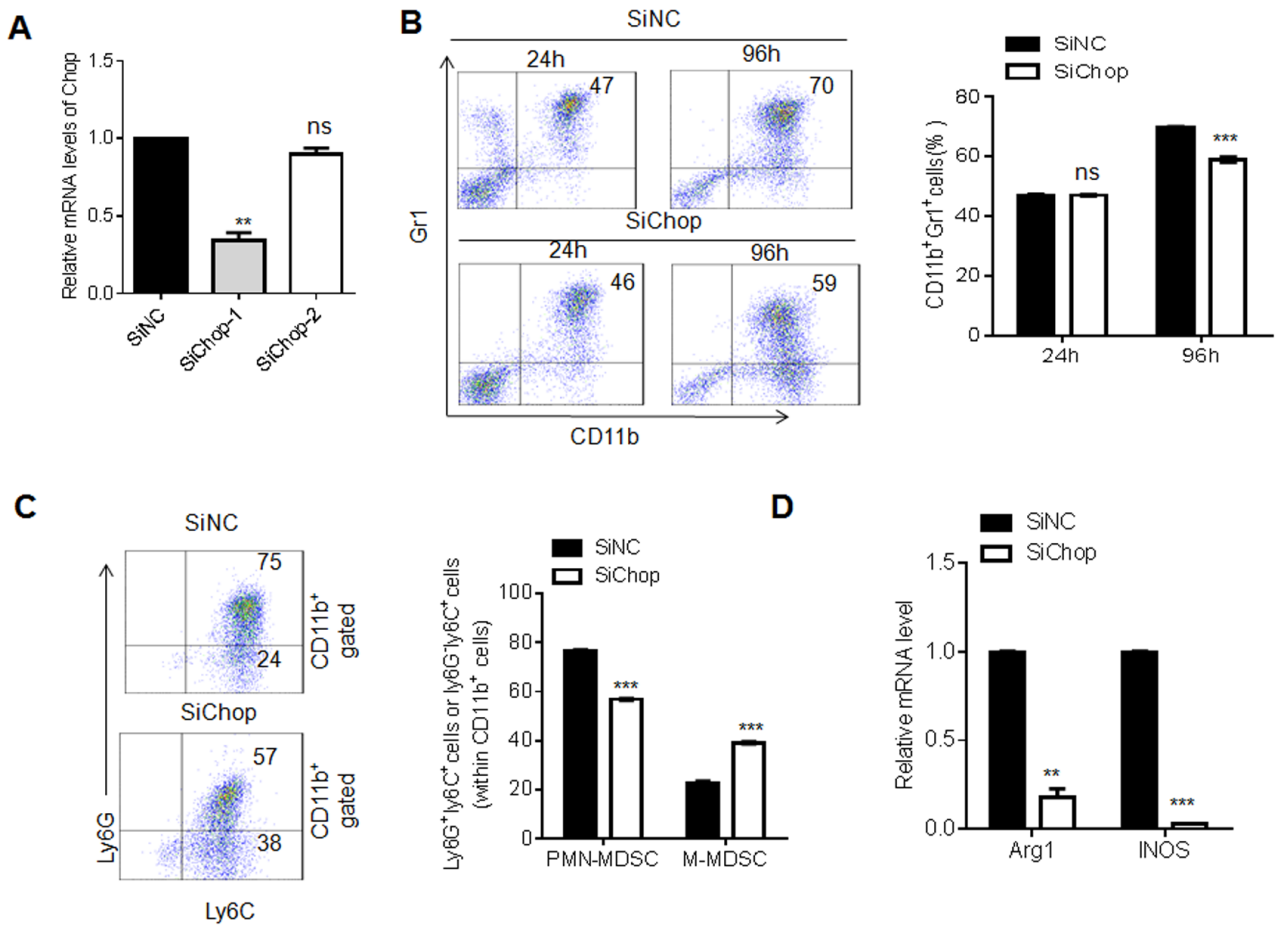
Chop knockdown exerts the same effect to miR-185-5p mimics during MDSC differentiation **(A)** QRT-PCR of Chop in MDSCs transfected with siNC (control siRNA), siChop-1(Chop siRNA 1) or siChop-2 (Chop siRNA 2). **(B)** Flow cytometric and statistical analyses in CD11b^+^Gr1^+^MDSCs transfected with siNC or siChop. **(C)** Flow cytometric and statistical analyses of MDSC subpopulations in MDSCs transfected with siNC and siChop. **(D)** QRT-PCR of Arg1 and iNOS in MDSCs transfected with siNC and siChop. The data are representative of at least three separate experiments. ^*^P < 0.05, ^**^P < 0.01, ^***^p<0.005; Ns, no significance.

Chop is the target of miR-185-5p, and miR-185-5p regulates MDSC differentiation, so we proposed that Chop knockdown should exert the same effect on MDSC differentiation with miR-185-5p mimics. Previous study show that Chop may promote differentiation and function of MDSCs *in vivo* [30]. We also found that Chop knockdown resulted in a decrease in the percentage of CD11b^+^Gr1^+^MDSCs as compared to control (Figure 7A and 7B). Furthermore, Chop knockdown also caused a significant decrease in the proportion of PMN-MDSCs and increase in M-MDSCs (Figure 7C). Meanwhile, qPCR revealed that Chop knockdown decreased the expression of Arg-1 and iNOS (Figure 7D). These results show that the effects of miR-185-5p on MDSCs are through targeting Chop.

### Regulation of RNCR3 in MDSCs is through upregulating Chop by sponging miR-185-5p

**Figure 8:**
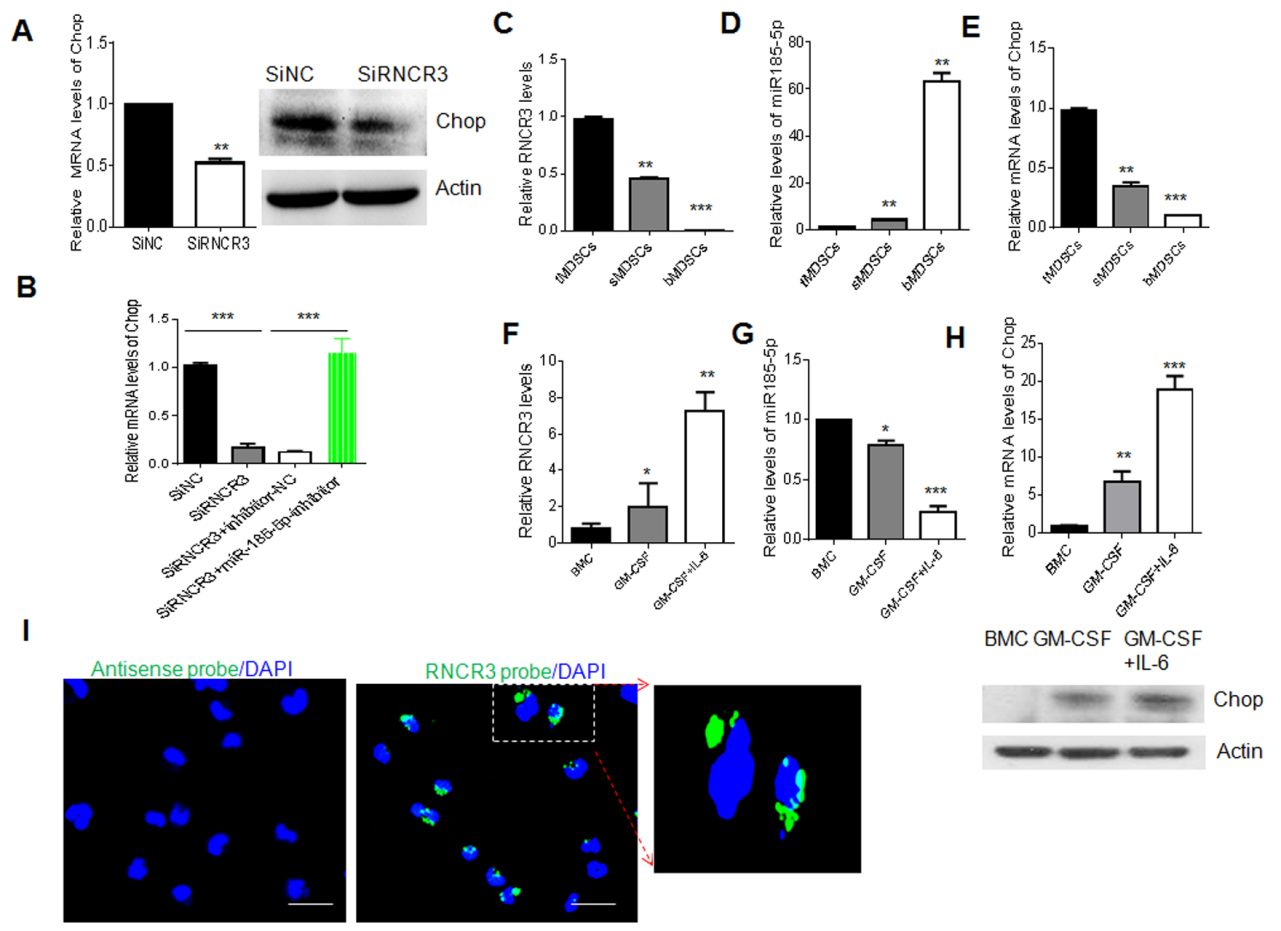
Regulation of RNCR3 in MDSCs is through upregulating Chop by sponging miR-185-5p **(A)** QRT-PCR (left) and Western blot (right) of Chop in MDSCs transfected with siRNA control (SiNC) and RNCR3 siRNA (SiRNCR3). **(B)** qRT-PCR of Chop in MDSCs transfected with RNCR3 siRNA and miR-185-5p inhibitor (siRNCR3+miR-185-5p) or miR-185-5p inhibitor control (siRNRC3+NC). siNC, siRNA control. **(C, D, E)** QRT-PCR of RNCR3, miR-185-5p and Chop in the MDSCs isolated from the tumor tissues (tMDSCs), spleen (sMDSCs) and BM (bMDSCs) of mice bearing B16 tumor. **(F, G, H)** QRT-PCR of RNCR3, miR-185-5p and Chop in CD11b^+^Gr1^+^BMCs (BMC), GM-CSF induced MDSCs (GM-CSF) and GM-CSF plus IL-6 mediated MDSCs (GM-CSF+IL-6). Western blot of Chop (lower in H). **(I)** RNA-FISH in the isolated MDSCs from the spleens of mice bearing B16 tumor. Green, RNCR3; Blue, nuclei. Rule bar = 20 μM. The data are representative of at least three separate experiments. ^*^P < 0.05, ^**^P < 0.01, ^***^p<0.005; Ns, no significance.

We finally investigated the relationship among lncRNA RNCR3, miR-185-5p and Chop in MDSCs. Since RNCR3 could act as ceRNAs interacting with miR-185-5p during MDSC differentiation, and Chop is a target of miR-185-5p, it is possible for RNCR3 to affect the expression of Chop. Indeed Chop was significantly downregulated in MDSCs transfected with RNCR3 siRNA as compared to control (Figure 8A). Importantly, Chop downregulated by silencing RNCR3 in MDSCs was rescued by miR-185-5p inhibitor (Figure 8B), indicating that RNCR3 positively regulates Chop expression by inhibiting miR-185-5p. To further illustrate the relationship among RNCR3, miR-185-5p and Chop, we examined the expression of RNCR3, miR-185-5p and Chop in differently isolated MDSCs (Figure 8C-8E), and different factors mediated MDSCs (Figure 8F-8H). While the level of RNCR3 expression was high, miR-185-5p was low and Chop was high; Conversely, while RNCR3 was low, the expression of miR-185-5p was high and Chop’s level was low (Figure 8C-8H). Since both RNCR3 and Chop RNAs are targets of miR-185-5p, it also is necessary to determine whether miR-185-5p has higher affinity to RNCR3 than to Chop mRNA. To test this, we first detected the Chop mRNA and RNCR3 expression dynamics in response to miR-185-5p transfection. While MDSCs from bone marrow were transfected miR-185-5p mimics, the levels of Chop and RNCR3 were both downregulated in a time-dependent and dose-dependent manner; but the alterations of RNCR3 was much remarkable than Chop, implying that miR-185-5p may have higher affinity to RNCR3 than to Chop mRNA (Supplementary Figure 4A and 4B). To further prove this, we employed dual luciferase report assays. RNCR3 WT (wild-type) and MUT (mutated) dual-luciferase reporter were constructed. While 293T cells were transfected with miR-185-5p mimics, luc-RNCR3 had lower luciferase activity than luc-Chop, indicating that miR-185-5p has higher affinity to RNCR3 (Supplementary Figure 4C-4E). Finally, RNA fluorescence *in situ* hybridization also showed that RNCR3 located not only in the nucleus but also in the cytoplasm of MDSCs (Figure 8I), further indicating the possibility for RNCR3 to bind with miR-185-5p in the cytoplasm. Taken together, our data suggest a RNCR3/miR-185-5p/Chop autologously strengthening pathway for the differentiation and immunosuppressive function of MDSCs.

## DISCUSSION

In this study we first found that MDSCs may express lncRNA RNCR3, and the expression is upregulated by tumor associated factors. We demonstrate that RNCR3 may promote MDSC differentiation and immunosuppressive function of MDSCs by binding to miR-185-5p and thereby releasing Chop. Since the levels of RNCR3 expression in MDSCs may be regulated by tumor-associated factors, this may disclose how MDSCs autologously strengthen differentiation and function of MDSCs in the tumor environment. Thus, while tumor-associated factors promote differentiation and immunosuppressive function of MDSCs, the upregulated RNCR3 may further promote the development of these processes.

More and more evidences have shown that lncRNAs play important roles in directing the development and differentiation of diverse immune cells. Multiple lincRNAs have been identified in myeloid immune cells such as that lncRNA HOTAIRM1 is reported to regulate the differentiation of myeloid cells [21]; Morrbid regulates the lifespan of short-lived myeloid cells [22]; Lnc-DC regulates differentiation of human dendritic cells [23] and lincRNA-Cox2 can turn on and off distinct classes of immune genes by different protein partners [31]. However, the effect of lncRNA on MDSCs is still absence of reports. We here demonstrate that lncRNA RNCR3 not only promotes the differentiation of MDSCs but also strengthens the immunosuppressive function of MDSCs.

LncRNAs regulate cell differentiation by multitudinous mechanisms such as lncRNAs can function as a ceRNA by sponging microRNAs or function as a guide, scaffold or decoy molecule by interacting with its protein partner [32]. LncRNAs bind to and sequester specific miRNA(s) to protect the target mRNAs from repression, which are revealed in many processes, including cell differentiation, tumorigenesis and pluripotency [33]. MicroRNA, a post-transcriptional regulator can repress target gene expression by binding to its complementary sequences in 3’ untranslated regions (3’UTRs) [34]. CeRNAs usually own miRNA-response elements and prevent the corresponding mRNAs from being degraded by competing for miRNAs. LncND, which contains 16 microRNA response elements for miR-143-3p, regulates the expression of Notch-1 and Notch-2 by sequestering miR-143-3p [35]. HOTAIR is reported to act as a ceRNA by sponging miR-613 and then suppresses its expression, leading to affect the expression of notch3 in pancreatic cancer [36]. RNCR3 functions as a ceRNA by sponging miR-185-5p and regulates the levels of KLF2 in endothelial cells [28]. We here also found that RNCR3 may functions as a ceRNA to promote Chop expression by sponging miR-185-5p during MDSC differentiation.

Chop is a cellular stress sensor C/EBP-homologous protein, which is induced by tumor-linked reactive oxygen and nitrogen species. Chop plays an important role in regulating the accumulation and immunosuppressive function of MDSCs. Chop-deficient MDSCs may display reduced signaling through CCAAT/enhancer-binding protein-β (cEBPβ) and reduced phospho-STAT3 [30]. Previous studies have shown that cEBPβ and STAT3 are critical transcription factors in regulating the differentiation and function of MDSCs [37–39]. Ablation of STAT3 expression through the use of conditional knockout mice or selective STAT3 inhibitor markedly reduces the expansion of MDSC and increased T cell responses in tumor-bearing mice [40, 41]. Recent studies also found that the generation of Ly6C^-^ monocytes involves induction of the transcription factor C/EBPβ and C/EBPβ-deficient mice lack Ly6C^-^ monocytes [38]. MDSCs lacking Chop have also decreased immune-regulatory functions and show the ability to prime T cell function and induce antitumor responses [30]. Our results also exhibit that Chop knockdown may affect the differentiation of MDSCs and decrease the levels of Arg-1 and iNOS, which plays a critical role in inhibiting T cell immunity. Meanwhile, we also found that Chop is significantly regulated by miR-185-5p, which can target multiple targeting molecules such as KLF2 or ABCC1 [28, 42]. Since miR-185-5p may be sponged by lncRNA RNCR3 during MDSC differentiation, we disclose a RNCR3/miR-185-5p/Chop regulatory network for strengthening differentiation and function of MDSCs.

MDSCs are immunosuppressive cells, which can promote tumor growth, differentiation and metastasis in a variety of ways [3]. Our studies show that adoptive transfer of MDSCs transfected with RNCR3 siRNA significantly reduces the generation of MDSCs, weakens the inhibitory function of MDSCs and suppresses tumor growth. Since RNCR3 expression may be regulated through tumor environment and inflammatory factors, our data suggest a new potential therapeutic target in cancer.

## MATERIALS AND METHODS

### Mice and cells

C57BL/6 and BALB/c mice were purchased from Beijing Animal Center (Beijing, China) and maintained in a specific pathogen-free (SPF) and controlled environment. B6.129S6-Il-6^tm1Kopf^ (IL-6^-/-^) and CD45.1 mice were purchased from Model Animal Research Center of Nanjing University (MARC, Nanjing, Jiangsu, China). OT-I transgenic mice were provided by Dr. Linrong Lu in Zhenjiang University. Experiments were carried out using age- and gender- matched mice. All the procedures were conducted according to Institutional Animal Care and Use Committee of the Model Animal Research Center. Animal experiments were approved by the Institute’s Animal Ethics Committee of Nankai University.

Murine melanoma B16 was obtained from American Type Culture Collection (ATCC, Manassas, VA, USA). Mosec ovarian cancer cells were gifted from Richard B. S. Roden (Johns Hopkins University, Maryland, USA). All of the cells were cultured in RPMI-1640 with 10% FBS (Hyclone, Logan, UT, USA) at 37°C in a humidified 5% CO_2_ atmosphere. CD11b^+^ Gr1^+^cells were sorted by FAScan or isolated using CD11b and Gr1 MACS MicroBeads and cell isolation kit (Miltenyi Biotec, Bergisch Gladbach, Germany) according to the manufacture’s instructions.

For *in vitro* induction of MDSCs, BMCs were obtained from the femurs of C57BL/6 mice and cultured in RPMI-1640 medium supplemented with GM-CSF (40 ng/ml) only or GM-CSF (40 ng/ml) plus IL-6 (40 ng/ml) for 4 days.

### Preparation of MDSCs tranfected by microRNA mimics, inhibitor and siRNA

Murine bone marrow cells were first transfected with control siRNA, RNCR3 siRNA, Chop siRNA, miR-185-5p mimics, miR-185-5p inhibitor or normal control using HiPerFect transfection reagent (Qiagen, Valencia, CA, USA) according to the manufacturer’s instructions, and then cultured for 4 days in the presence of GM-CSF or GM-CSF plus IL-6. All microRNA, siRNAs and control siRNAs were purchased from Riobio (Guangzhou, China). The target sequences for RNCR3 siRNAs: siRNA1, 5’- GGATGCGGGAGAACAAAGA-3’ and siRNA 2, 5’- TCACAGCGGACCTTGATTT-3’; The target sequences for Chop siRNAs: siRNA 1, 5’- CCTAACACGTCGATTATAT-3’; and siRNA 2, 5’- GCTCTCCAGATTCCAGTCA-3’.

### Construction and transduction of RNCR3 shRNA/lentivirus

A shRNA target (5’- TCACAGCGGACCTTG ATTT-3’) was chosen from the target sequences produced by BLOCK-iT™ RNAi Designer (Invitrogen) and/or by i-Score Designer38. The shRNA constructs were made using pGreenPuro™ shRNA Cloning and Expression Lentivector kit (System Biosciences Inc.) according to the manual. The control shNC is the Luciferase Control shRNA from the kit. For packaging of lentivirus particles, the shRNA lentivector together with pMD2.G and psPAX2 packaging plasmids were cotransfected into 293T cells.

MDSCs were infected with the lentiviral supernatants in the presence of 8μg/ml polybrene (Millipore) by centrifugation and then cultured with complete medium for 24 hours. The cells were then washed and cultured under GM-CSF or GM-CSF plus IL-6 for 4 days.

### *In vitro* MDSC suppression assay

To measure immunosuppressive function of different treated MDSCs, MDSCs and splenocytes obtained from OT-I mice were co-cultured with 200 nM OVA peptide (OVA257-264) in 96-well plates at a ratio of 1:0,1:1,1:4 and 0:1 for 48h. The production of IFN-γ was measured by ELISA Kit (4A Biotech, Beijing, China) according to the manufacturer’s instructions.

### Reactive oxygen species and nitric oxide detection

Oxidation-sensitive dye DCFDA (Diacetyldichlorofluorescein, Molecular Probes/Invitrogen) was used to measure reactive oxygen species (ROS) production by MDSCs according to the reported protocol [43]. Isolated CD11b^+^Gr1^+^MDSCs were simultaneously cultured with 2.5 μM DCFDA and 30 ng/ml phorbol myristate acetate (PMA, Sigma-Aldrich) for 30 min. Analyses were then conducted by flow cytometry as described above.

For nitric oxide production, the total nitric oxide in the cell lysate was measured using the Nitrate/Nitrite Assay Kit. Equal volumes of cell lysate (60 μl), 2mM NADPH (5 ul), FAD (10ul) and Nitrate Reductase (5ul) were incubated at 37°C for 30 min, followed by addition of 10 ul of LDH Buffer and LDH solution. After incubation for 30 min at 37°C. Then, addition of 50 ul of Griess Reagent I and Griess Reagent II, incubate at room temperature for 10 minutes and absorbance at 540 nm was measured. Nitrite concentrations were quantified by comparing the absorbance values to a standard curve generated by serial dilution of 100 uM sodium nitrite.

### *In vivo* experiments

C57BL/6 B16 melanoma mouse model was used to investigate the effect(s) of RNCR3-modified MDSCs on tumor growth. Mice were injected with 1×10^6^ B16 and were randomly divided into several experimental groups (six mice/group), and then the prepared MDSCs (1×10^6^) were injected into different groups via tail vein at day 5, day 11 and day 18 after injection of tumor cells. For the preparation of RNCR3-modified MDSCs, BMCs obtained from C57BL/6 CD45.1 mice were first transfected with control siRNA or RNCR3 siRNA according to our previous method [44] or transduced by RNCR3 shRNA/lentiviruses and then cultured with GM-CSF (40 ng/ml) plus IL-6 (40 ng/ml) for 4 days. The tumor volume were measured in two dimensions by calipers every 2 days and calculated by the following formula: Width^2^×Length×π/6.

### RNA extraction and qRT-PCR

Total RNA was extracted from cells by using TRIzol reagent (Life Technologies, Carlsbad, CA) and was transcribed to cDNA using HiFiScript cDNA Synthesis Kit (CWBIO, Beijing, China) according to the manufacturer’s instructions. The quantitative real-time PCR (qRT-PCR) was performed by using Hieff^™^ qPCR SYBR-Green Master Mix (YEASEN, Shanghai, China) in a Bio-Rad iQ5 multicolour RT-PCR system. The primers used for qRT-PCR were showed in Supplementary Table 1. The levels of each gene was calculated using the 2^−ΔΔCT^ method. The stem-loop RT primer (5’- GTCGTATCCAGTGCAGGGTCCGAGGTATTCGCACTGGATACGACTCAGGA-3’) was used for miR-185-5p level detection. The relative expression level of miR-185-5p was normalized to that of internal control U6. For other genes, GAPDH was used as the endogenous control.

### Flow cytometric analyses

Cells were collected and washed twice with PBS, and then incubated in PBS with 1% FBS and antibodies for 30min. After washing twice with PBS, cells were fixed in 1% paraformaldehyde and analyzed by the FACScan flow cytometer (BD Biosciences). Anti–Gr1-FITC(RB6-8C5), CD11b-FITC(M1/70), Ly6G-PE(1A8), Ly6C-FITC(AL-21) and CD45.1-APC(A20) were purchased from BD Biosciences (San Diego, CA). Anti-CD11b-PercP/cy5.5(M1/70), Gr1-PE(RB6-8C5), CD45-APC(30-F11), CD4-PE(GK1.5), CD8a-FITC(53-6.7) and 7-AAD were purchased from BioLegend (San Diego, CA).

### Western blot

Western blot was performed as described previously [45]. Hybridizations with primary Abs were carried out for 1 hr at room temperature in blocking buffer. The protein-Ab complexes were detected using peroxidase-conjugated secondary Abs (Boehringer Mannheim) and ECL (Amersham Biosciences). Antibodies against Chop (Cell Signaling Technology, Boston, MA, USA, 1:2000 dilution), Arg-1(Santa Cruz Biotechnology, Santa Cruz, CA, 1:1000 dilution), iNOS (Santa Cruz Biotechnology, Santa Cruz, CA, 1:1000 dilution) and β-actin (Santa Cruz Biotechnology, Santa Cruz, CA, 1:1000 dilution) were used.

### RNA fluorescence *in situ* hybridization

RNA fluorescence *in situ* hybridization (RNA-FISH) was performed according to reported protocol [46]. The cells first grew to desired confluency on sterile, 0.01% poly-lysine-treated glass slips in the bottom of a 6-well tissue culture dish. Aspirate the media and pipette 2 ml of ice-cold CSK buffer into 6-well plate for 30s, 2 ml of ice-cold CSK + 0.4 % Triton X-100 buffer for 30s, 2 ml of ice-cold CSK buffer for 30s for cell membrane perforation. Pipette 2 ml of 4 % PFA for 10 min, washed with cold 70 % ethanol three times for cell fixation. The slides were dehydrated by moving them through a room temperature ethanol series (85, 95, and 100 % ethanol) for 2 min each, and air-dried at room temperature for 15 min and hybridized using the indicated probes overnight at 37°C in a humid chamber. After washing with 2× SSC/50 % formamide, 2× SSC, and 1× SSC each for three times, DAPI dye was added. Seal the slides and observed using confocal microscope. The probe sequences were showed in Supplementary Table 1.

### Dual luciferase assay

WT-3’UTR-Chop, WT-RNCR3, MUT(mutated)-3’UTR-Chop and MUT-RNCR3 plasmids were constructed by cloning WT and mutated 3’UTR sequence of Chop or WT and mutated RNCR3 sequence into downstream of firefly luciferase cassette in pSiCHECK-2 vector (Promega). The mutated sites of both Chop 3’UTR and RNCR3 were in miR-185-5p binding sites. The Chop 3′UTR and RNCR3 mutants were generated according to our previous method [47]. The primers used were listed in Supplementary Table 1. Mutations were confirmed by plasmid DNA sequencing. HEK293T cells were cultured in a 24-well plate at 1×10^5^ cells per well and then co-transfected with WT-3’UTR-Chop or MUT-3’UTR-Chop and miR-185-5p mimic or mimic control, or WT-RNCR3 or MUT-RNCR3 and miR-185-5p mimic or mimic control with using Lipofectamine 2000 (Invitrogen). After transfection for 48h, relative luciferase activity was calculated by normalizing Firefly luminescence to Renilla luminescence using a Dual-Luciferase Reporter Assay (Promega, Madison, USA) according to the manufacturer’s instructions.

### Statistical analyses

All quantitative data were expressed as mean ± SEM. A Student *t* test and one-way ANOVA were used to determine statistical differences. A 95% confidence interval was considered significant and was defined as *p*<0.05.

## SUPPLEMENTARY MATERIALS FIGURES AND TABLE




