# Supplementary material for: LncRNA RNCR3 promotes Chop expression by sponging miR-185-5p during MDSC differentiation
Source: Oncotarget. 2017 Dec 4;8(67):111754–69. doi: 10.18632/oncotarget.22906 (PMC5762357; doi:10.18632/oncotarget.22906)
Supplement: Supplementary file 1 [file oncotarget-08-111754-s001.pdf]

LncRNA RNCR3 promotes Chop expression by sponging miR-185-5p during MDSC differentiation

SUPPLEMENTARY MATERIALS

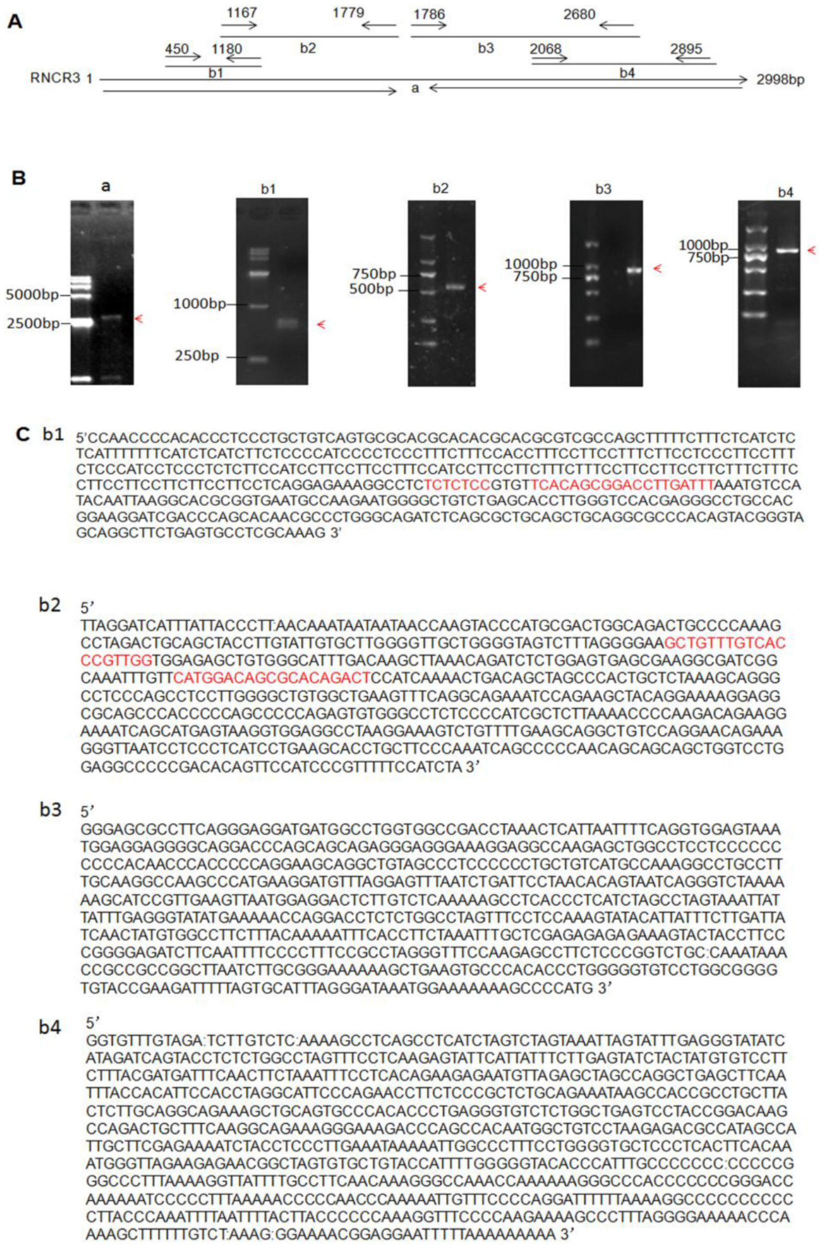

Supplementary Figure 1: RNCR3 expression in MDSCs. (A) The scheme indicating full-long RNCR3 and RNCR3 fragments, which was segmented and amplified. The arrows showed the location of different pairs of primers. (a) indicated the overall length of RNCR3; (b1-b4) indicated the fragments of amplification. The primers were listed in Supplementary Table 1. **(B)** Gel electrophoresis of different lengths of PCR products, which represented the corresponding fragments respectively in the scheme of (A). The DNA markers was showed in the left of each picture. Red arrow indicated the position of products. **(C)** Sequences of different PCR products from b1, b2, b3 and b4. The first red area in b1 showed the site of RNCR3 binding to miR-185-5p; The second red area in b1 indicated the target site of RNCR3 silencing (siRNCR3-2); The RT-PCR primers for RNCR3 were shown in the red area of b2.

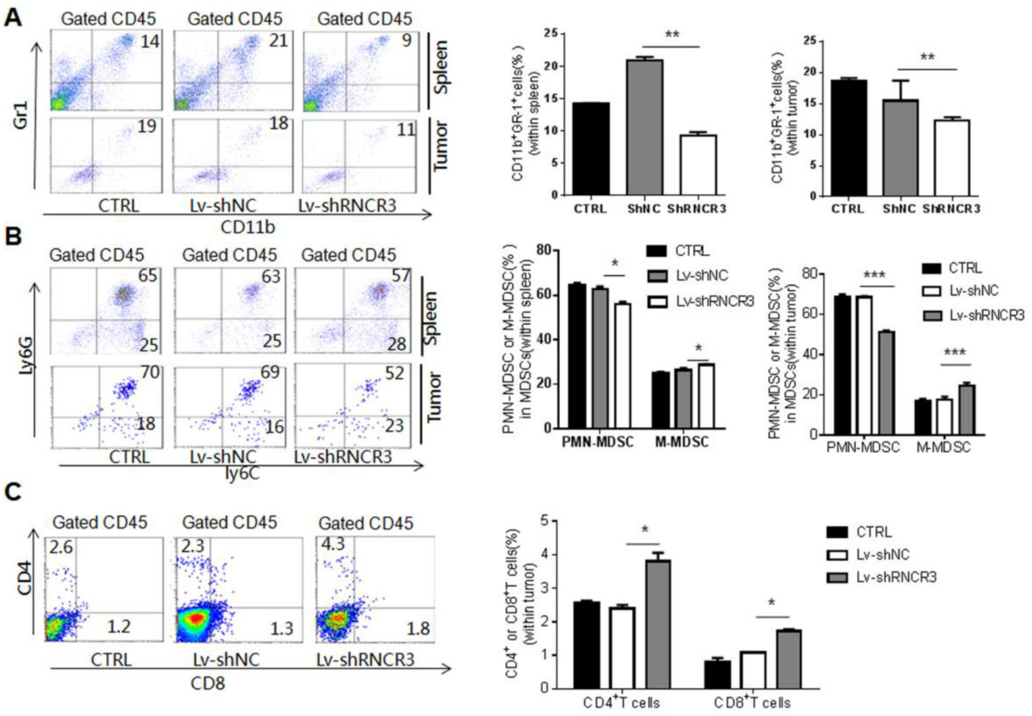

Supplementary Figure 2: Flow cytometric and statistical analyses of MDSCs **(A)** and their subpopulations **(B)** in the spleen and tumor from mice injected with CD45. 1+ MDSCs transfected with control lentivirus (Lv-shNC) or RNCR3 shRNA lentivirus (Lv-shRNCR3), and flow cytometric and statistical analyses of CD4+ or CD8+ cells in the tumor from mice injected with CD45.1+MDSCs transduced with control lentivirus (Lv-shNC) or RNCR3 shRNA lentivirus (Lv-shRNCR3) **(C)**. CTRL, without MDSC injection. The data are from three separate experiments. $^{*}P < 0.05$, $^{**}P < 0.01$, $^{***}p<0.005$; Ns, no significance.

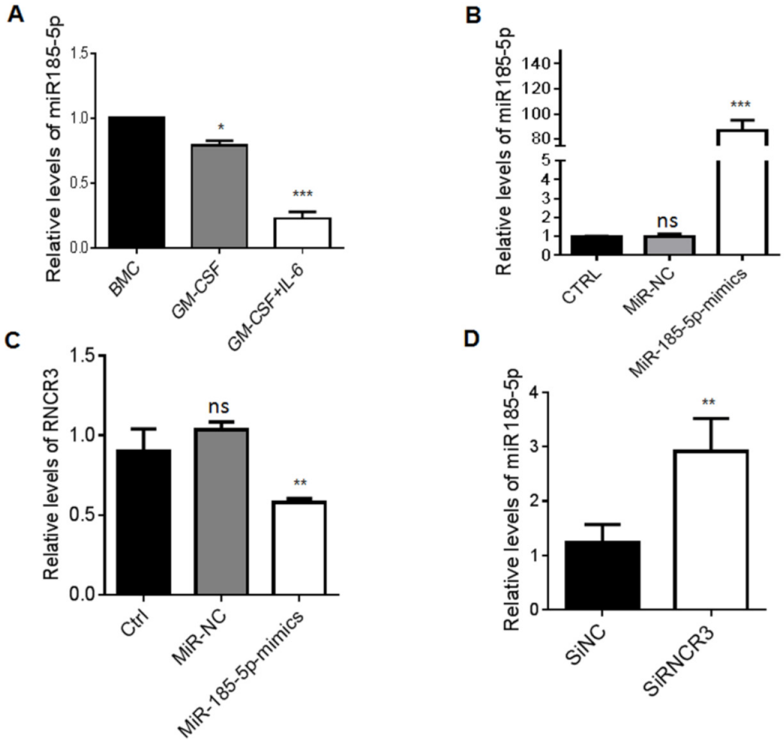

Supplementary Figure 3: A reciprocal interaction between lncRNA RNCR3 and miR-185-5p in MDSCs. **(A)** QRT-PCR of miR-185-5p in MDSCs induced by GM-CSF or GM-CSF plus IL-6. Fresh Gr1^{+}CD11b^{+}BMCs were used as control. **(B** and **C)** QRT-PCR of miR-185-5p (C) and RNCR3(D) in MDSCs transfected with miR-185-5p mimics or mimics control (MiR-NC) in the presence of GM-CSF and IL-6. Fresh Gr1^{+}CD11b^{+} BMCs were used as control. **(D)** QRT-PCR of miR-185-5p in the MDSCs transfected with siRNA control (SiNC) or RNCR3 siRNA (SiRNCR3) in the presence of GM-CSF and IL-6. The data are from three separate experiments. $^{*}P < 0.05$, $^{**}P < 0.01$, $^{***}p<0.005$; Ns, no significance.

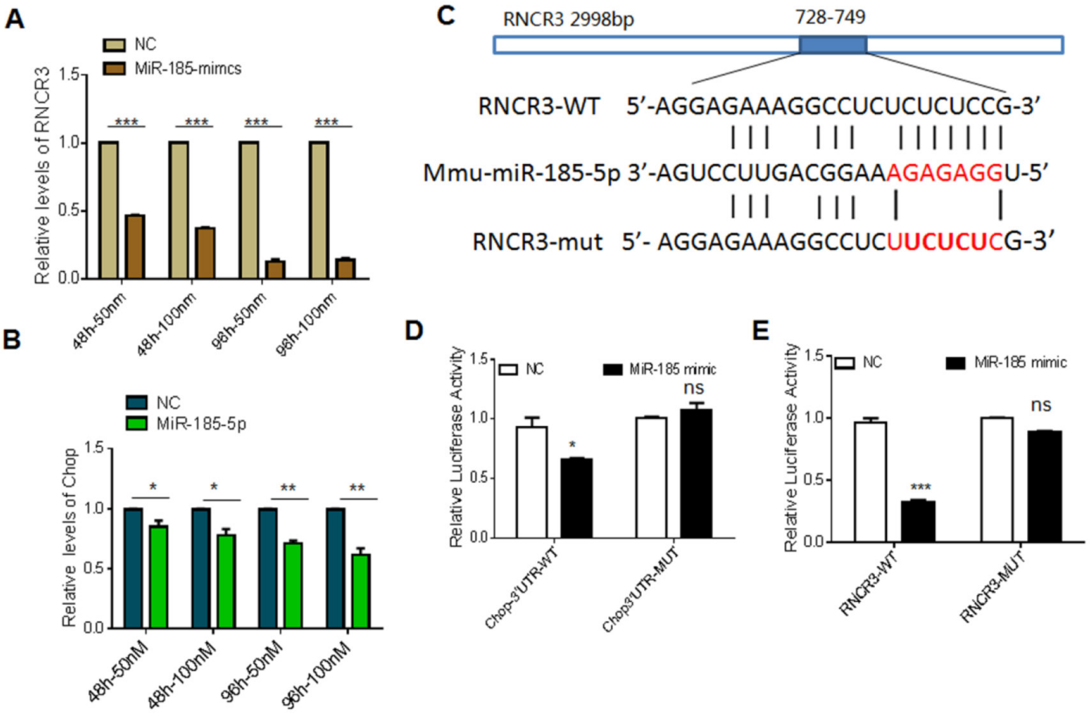

Supplementary Figure 4: miR-185-5p has higher affinity to RNCR3 than to Chop. (**A** and **B**) QRT-PCR of RNCR3 (A) and Chop (B) in miR-185-5p transfected MDSCs at the indicated time and indicated dose. **(C)** Potential site targeted by miR-185-5p in RNCR3 sequence and the mutant site (red) of RNCR3 in pSiCHECK™-2 luciferase reporter vector. **(D)** Dual-luciferase reporter assay of 293T cells co-transfected with Chop 3’-UTR-WT or Chop 3’-UTR-Mut and miR-185-5p mimics or mimics control (NC). **(E)** Dual-luciferase reporter assay of 293T cells co-transfected with RNCR3-WT or RNCR3-Mut and miR-185-5p mimics or mimics control (NC). The data are representative of at least three separate experiments. $^{*}P < 0.05$, $^{**}P < 0.01$, $^{***}p<0.005$; Ns, no significance.

Supplementary Table 1: Oligoes used in this study.

See Supplementary File 1
