# Supplementary material for: LncRNA RNCR3 promotes Chop expression by sponging miR-185-5p during MDSC differentiation
Source: Oncotarget. 2017 Dec 4;8(67):111754–69. doi: 10.18632/oncotarget.22906 (PMC5762357; doi:10.18632/oncotarget.22906)
Supplement: Supplementary file 2 [file oncotarget-08-111754-s002.docx]

**Supplementary Table 1: Oligoes used in this study**

| Oligo name | Sequence (5’ to 3’) | |
| --- | --- | --- |
| Murine GAPDH-s  Murine GAPDH-as | GGTGAAGGTCGGTGTGAACG  CTCGCTCCTGGAAGATGGTG | |
| Murine Chop -s  Murine Chop-as | GTCACACGCACATCCCAAAG  CACTTTCCGCTCGTTCTCCT | |
| MiR-185-5p-s  MiR-185-5p-as | GGACGCGTGGAGAGAAAGG  GTATCCAGTGCAGGGTCCGAGGT | |
| Murine U6-s  Murine U6-as | CTCGCTTCGGCAGCACA  AACGCTTCACGAATTTGCGT | |
| Murine Arg1 -s  Murine Arg1-as | CTGACCTATGTGTCATTTGGG  TCAGGAGAAAGGACACAGGTT | |
| Murine iNOS -s  Murine iNOS-as | AGGAGGAGAGAGATCCGATTT  GCTTGTCACCACCAGCAGTAG | |
| RNCR3 a-s  RNCR3 a-as | TCACCCAACGAGGACAGT  AACCGCAGGCACCATCCATA | |
| RNCR3 b1-s  RNCR3 b1-as | GAGCTAAGGACCACCGCATC  CGACACCTTCATCCCTACCTG | |
| RNCR3 b2-s  RNCR3 b2-as | CATTGTAGAAGGCCGACACC  AGATGGAGACGGGATGGAAC | |
| RNCR3 b3-s  RNCR3 b3-as | TCCCGTCTCCATCTCAGTGT  ACAGCACACTAGCCGTTCTC | |
| RNCR3 b4-s  RNCR3 b4-as | ATCAGGGTCTAAAAGAGCATCCG  ATGGATGGTGCCTGCGGTTT | |
| Murine RNCR3-s  Murine RNCR3-as | GCTGTTTGTCACCCGTTGG  CATGGACAGCGCACAGACT | |
| Chop-Xho1-a  Chop-Not1-as | CCGCTCGAGACAGTGGGCATCACCTCC  ATTTGCGGCCGCAGTCACTTTACTGGACATGGAC | |
| chop-MUT-as  chop-MUT-a | GTACACTTCCCCTCTCTCAGACAGGAG  TGTCTGAGAGAGGGGAAGTGTACCCAG | |
| RNCR3-Xho1-a  RNCR3-Not1-as | CCGCTCGAGGATGCGGTGGTCCTTAGCTC  ATTTGCGGCCGCCAGGTAGGGATGAAGGTGTCG | |
| RNCR3-MUT-as  RNCR3-MUT-a | CTGTGAACACGAGAGAAGAGGCCTTT  GGAGAAAGGCCTCTTCTCTCGTGTTC | |
| Probes sequence in RNA-FISH | | |
| RNCR3-FAM | | FAM-CTCTCGCTCAGAGGTCCGCAG |
| Antisense-FAM | | FAM-CTGCGGACCTCTGAGCGAGAG |
